# Sexual transmission of Hepatitis C virus between HIV infected subjects and their main heterosexual partners; Actor Partner Interdependent Modelling

**DOI:** 10.1186/1471-2334-14-S2-P87

**Published:** 2014-05-23

**Authors:** Abbas Alipour, Abbas Rezaianzadeh, Jafar Hasanzadeh, Abdorreza Rajaeefard, Mohammad Ali Davarpanah

**Affiliations:** 1 Social medicine department, Medical school, Mazandaran university of medical sciences, Sari, Iran; 2 Epidemiology department, Health and Nutrition School, Shiraz university of medical sciences, Sari, Iran; 3 HIV Research center, Shiraz University of medical Sciences, Sari, Iran

## Introduction

Overall, 60-70% of the hepatitis C virus’ (HCV) transmission routes is parenteral and in 30-40% of the cases was unknown (e.g. sexual route). Knowing of these routes in HIV infected dyads very important due to clinical and methodological reasons. Whether HCV transmits through sexual route in these dyads is controversy.

## Materials and methods

Of the 1338 dyads enrolled in Behavioural Consultation Center in Shiraz, Iran in 2011, 984 couples (HIV infected subjects and their main heterosexual partners) have eligibility criteria. Random samples, 168 of 984 couples, were chosen through random generated numbers using a computer program. We used actor partner independent model (APIM) and multilevel analysis to assess multiple risk factors for HCV while partitioning the source of risk at the individual and couple level.

## Results

Age of the index samples was 38.71± 7 years and 33.2± 6.3 years for their main heterosexual partners; the mean duration of sexual relationship for couples was 11.9 (median= 8.5) years. Multivariate analysis showed that actor risk factor of intravenous drug using (IDU) (AOR= 13.03; 95% CI: 3.9- 43.82) and actor cofactors of HIV positivity (AOR= 7.1; 95% CI: 1.37- 36.97), razor sharing (AOR= 4.81; 95% CI: 1.84- 12.55), sex (AOR= 8.83; 95% CI: 3.16- 24.87), and condom use in sexual activity with main partner (AOR= 0.15; 95% CI: 0.02- 0.44) were associated with actor HCV positivity.

## Conclusion

Health care providers need to pay special attention to sexual transmission of HCV among HIV-infected individuals and the control/preventive measures for HCV sexual transmission should be recommended.

**Table 1:** Multilevel logistic regression estimation of individual and couple level risk factors for actor anti-HCV positivity, final model, Shiraz (southern of Iran), 2011

Effects^†^	β	SE	AOR (95% CI)
**Actor IDU**	2.57	0.62	13.03 (3.9- 43.82)

**Actor Razor sharing**	1.57	0.49	4.81 (1.84- 12.55)

			

**Actor HIV positive**	1.96	0.84	7.1 (1.37- 36.97)

**Actor male**	2.18	0.52	8.83 (3.16- 24.78)

**No condom use in sexual activity with main heterosexual partner**	**1.89**	**0.92**	**6.6 (1.1- 39.6)**

^†^**Random effect (level 2 variance component), σ_u_**= 3.98^-19^

